# The translation and validation of the YouTube addiction scale in modern standard Arabic among emerging and young adults

**DOI:** 10.1007/s44192-026-00498-1

**Published:** 2026-06-22

**Authors:** Haitham Jahrami, Khaled Trabelsi, Waqar Husain, Hadeel Ghazzawi, Zahra Saif, Achraf Ammar, Amir H. Pakpour

**Affiliations:** 1 Government Hospitals, Manama, Bahrain; 2 Department of Psychiatry, College of Medicine and Medical Sciences, Arabian Gulf University, Manama, Bahrain; 3 Research Laboratory Education, Motricité, Sport et Santé, EM2S, LR19JS01, High Institute of Sport and Physical Education of Sfax, University of Sfax, Sfax, Tunisia; 4 Department of Movement Sciences and Sports Training, School of Sport Science, The University of Jordan, Amman, Jordan; 5 Department of Humanities, COMSATS University Islamabad, Islamabad Campus, Park Road, Islamabad, Pakistan; 6 Nutrition and Food Technology Department, Agriculture School, The University of Jordan, Amman, Jordan; 7 Department of Training and Movement Science, Institute of Sport Science, Johannes Gutenberg-University Mainz, 55099 Mainz, Germany; 8 Research Laboratory, Molecular Bases of Human Pathology, LR19ES13, Faculty of Medicine of Sfax, University of Sfax, 3000 Sfax, Tunisia; 9 Department of Nursing, School of Health and Welfare, Jönköping University, Jönköping, Sweden; 10 Division of Anesthesia, Operation, and Intensive Care, Södertälje Hospital, Södertälje, Sweden

**Keywords:** Behavioral addiction, Psychometric validation, Social media addiction, YouTube addiction scale, YouTube addiction

## Abstract

**Background:**

The widespread use of YouTube has raised concerns about its potential for addiction, particularly in Arabic-speaking populations where social media consumption is prevalent. A culturally tailored tool to assess YouTube addiction is essential for effective research and intervention in these communities.

**Objective:**

This study aimed to translate and validate the 6-item YouTube Addiction Scale (YAS) into Arabic, ensuring its psychometric robustness for assessing YouTube addiction among Arabic-speaking emerging and young adults.

**Methods:**

A cross-sectional study was conducted with 1,134 Arabic-speaking emerging and young adults from Bahrain, Saudi Arabia, Jordan, and Tunisia recruited through convenience sampling on social media platforms. The YAS was translated via the forward‒backward‒forward technique. The psychometric evaluation included confirmatory factor analysis (CFA), item response theory (IRT), reliability analyses (McDonald’s *ω*, Cronbach’s *α*, and composite reliability [CR]), and test-retest reliability. Convergent and divergent validity were assessed through correlations with the Insomnia Severity Index (ISI), Modified Yale Food Addiction Scale (mYFAS), Depression Anxiety Stress Scale (DASS-21), and Bergen Social Media Addiction Scale (BSMAS).

**Results:**

The Arabic YAS demonstrated a unidimensional structure with adequate factor loadings (0.55–0.73). The model fit indices were excellent (CFI = 0.99, TLI = 0.98, RMSEA = 0.06, *χ²*(9) = 40.66, *p <* 0.001), with good internal consistency (*ω* = 0.81, *α* = 0.80, CR *=* 0.80) and test-retest reliability (ICC = 0.87). IRT analysis confirmed item fit (infit/outfit 0.86–1.17) and person reliability (0.78). Significant correlations with the total score of BSMAS (*r =* 0.66), DASS-21 (*r =* 0.40), mYFAS (*r =* 0.32), and ISI (*r =* 0.26) supported validity. Measurement invariance was confirmed across gender and weekly YouTube use. Scalar invariance was also supported across age groups (18–21 vs. 22–25 years).

**Conclusions:**

The Arabic YAS is a psychometrically sound tool for assessing problematic YouTube use among Arabic-speaking emerging and young adults, enabling researchers and clinicians to screen for elevated risk in this high-engagement developmental stage. Further studies should examine age-related differences within and beyond emerging adulthood, as well as longitudinal patterns of use and associated outcomes in this population.

## Introduction

In the contemporary digital landscape, the widespread consumption of online content has led to the emergence of YouTube addiction as a significant concern [1, 2]. The pervasive nature of social media, including video-sharing platforms such as YouTube, is increasingly recognized as a source of technological addiction across adolescent and adult populations globally [2, 3]. Overuse of social media has been consistently linked to various adverse outcomes [4, 5], such as diminished work performance [5, 6], less healthy social relationships [7–9], sleep disturbances [10–13], reduced life satisfaction [10, 14], and heightened feelings of jealousy [15, 16], anxiety [17, 18], and depression [19, 20]. To effectively understand and address this burgeoning phenomenon, accurate and culturally appropriate assessment tools are crucial.

Understanding the full spectrum of YouTube addiction, ranging from mild preoccupation to severe dependence, necessitates specialized instruments [1, 2]. While general measures of internet addiction (IA) [21] and social media addiction (SMA) [22] exist, YouTube has unique architectural and functional characteristics that distinguish it from other social networking sites [1]. Unlike social networking sites (SNSs), which focus primarily on user-to-user interactions and personal profiles, YouTube is predominantly a content community centered on video viewing [1]. Its design, including personalized content recommendations and the allure of constant novelty, creates a highly personalized digital experience that can be difficult to resist, thereby contributing to its addictive potential [1]. This distinct nature underscores the need for specific tools, such as the YouTube Addiction Scale (YAS), to provide in-depth information for clinical decision making and research [1]. The YAS, developed by experts in psychology and digital behavior, aims to quantify individuals’ levels of dependence on this popular video-sharing platform, assessing aspects such as time spent, impact on daily life, and ability to control usage. Individuals with high YAS scores may experience negative consequences, such as neglecting responsibilities, sleep disturbances, and strained relationships due to excessive consumption [1].

The prevalence of social media usage, particularly YouTube, is notably high across various demographics. For example, a recent study indicated that over 97% of respondents actively use social media, with YouTube being one of the most popular platforms, accessed predominantly via mobile phones (88.7%) [2, 23]. A significant proportion of users, nearly 38.7%, reported watching YouTube videos daily, with 21.7% spending over five hours a day [2]. Such extensive engagement highlights the potential for problematic use and underscores the global scale of this issue. Moreover, studies have shown that individuals with higher levels of YouTube addiction often exhibit greater general social media addiction and psychological distress, including depression, anxiety, and stress [1–3, 12]. The existence of monetization programs (e.g., the YouTube Partner Program [YPP]) also creates a competitive environment among content creators, which can further contribute to addictive engagement and excessive use due to the desire for success and revenue [24]. These factors emphasize the urgent need for robust measurement instruments to identify and understand problematic YouTube usage [1].

A comprehensive meta-analysis of 504 studies covering 2,123,762 individuals from 64 countries estimated the pooled prevalence of SMA at 17.42% (95% CI 12.42–23.89) and IA at 14.22% (95% CI 12.90–15.65) in the general population, with rates of problematic digital engagement rising markedly in recent years [10]. In the Arab world, where internet penetration reaches 77%—well above the global average—and young people maintain an average of 8.4 social media accounts while spending more than 3.5 h daily online [25, 26], the issue is particularly pronounced. Recent regional surveys indicate that 74% of Arab youth struggle to disconnect from social media platforms, with 61% explicitly acknowledging that such addiction negatively affects their mental well-being [25, 26]. Moreover, Saudi Arabia alone leads the Arab world in per capita YouTube consumption, where 68% of users watch more video content digitally than on traditional television [25, 26].

Given the significant impact of cultural and linguistic elements on the perception and treatment of psychological constructs, including addiction, there is a crucial need for culturally tailored and validated assessment tools. Arabic is one of the most prevalent languages worldwide, spoken by more than 450 million people, making the availability of such instruments vital for accurate assessment and effective intervention in Arab societies [27]. While existing measures, such as the Bergen Social Media Addiction Scale (BSMAS) have been validated in different language versions, including Arabic [28], they assess general social media addiction and may not capture the specific nuances of YouTube addiction. Although previous efforts have translated and validated instruments to assess addictive behaviors in Arabic-speaking populations [28], a comprehensive tool specifically designed for YouTube addiction has been lacking. While general SMA scales, such as the BSMAS effectively capture broad problematic use across platforms, they fail to account for the unique, algorithm-driven, video-centric features of YouTube—such as passive binge watching, personalized recommendation loops, and extended viewing sessions—that may drive distinct patterns of addiction and warrant a platform-specific instrument.

Therefore, the translation and validation of the YAS into Arabic holds significant importance for several reasons. First, it will enable researchers and mental health professionals to gain a deeper understanding of how YouTube addiction manifests and interacts within Arab societies. Second, it will provide a psychometrically sound instrument to accurately assess problematic YouTube usage among Arabic-speaking individuals. Third, a validated Arabic YAS will facilitate investigations into the psychological and social consequences of these behaviors in the region. Finally, and perhaps most critically, it will support the development and implementation of evidence-based prevention and intervention strategies specifically tailored to Arab cultural contexts. This tool would allow healthcare providers to monitor the severity of individuals’ YouTube addiction and intervene early when necessary while also enabling researchers to explore new topics and promote cross-country comparisons.

The present study aims to address this critical gap by translating and validating the 6-item YAS [1], originally developed based on Griffiths’ component model of addiction, for use within Arabic-speaking populations. This endeavor employs rigorous psychometric testing methods to ensure that the YAS possesses strong internal consistency, unidimensionality, and construct validity for this linguistic and cultural context. While high levels of YouTube engagement can be functional, e.g., for education, entertainment, or relaxation without impairment, problematic use characterized by loss of control, continued use despite negative consequences, and interference with daily functioning has been increasingly documented [1, 2]. The present study focuses on assessing the severity of such problematic patterns via a dimensional tool without implying a formal clinical diagnosis.

## Methods

### Study design and setting

This study employed a cross-sectional correlational design, a widely recognized methodology for simultaneously assessing the prevalence of behavioral traits and evaluating the psychometric properties of instruments at a single point in time. The research was conducted among the emerging and young adult populations residing within four Arabic-speaking countries (i.e., Bahrain, Saudi Arabia, Jordan, and Tunisia). The concept of emerging and young adulthood was introduced by Jeffrey Jensen Arnett (2000) as a distinct developmental stage roughly spanning ages 18–25, characterized by identity exploration, instability, self-focus, feeling in between, and a sense of possibilities [29]. This period is particularly relevant to studies of technology use, behavioral addictions, and psychological transitions in modern contexts [30].

This multi-country approach was chosen to ensure direct cultural relevance and enhance the generalizability of findings to the broader Arabic linguistic group.

### Participants

Participants were recruited through a convenience sampling method, with the online questionnaire widely distributed via various social media channels, a technique proven effective in reaching diverse emerging and young adult populations for research purposes. The inclusion criteria specified Arabic-speaking emerging and young adults aged 18 years or older who were also residents of Bahrain, Saudi Arabia, Jordan, or Tunisia at the time of the survey. This age demographic was particularly pertinent, as young adulthood is recognized as a crucial developmental period marked by significant life transitions and the emergence of complex self-regulatory behaviors related to time management. Previous studies in Saudi Arabia have consistently shown a high representation of young adults, particularly students, in similar samples [31]. A total of 1,134 individuals participated in the study.

### Procedures

Data collection was managed through a secure online survey platform, which inherently ensured participant anonymity and voluntary participation. The comprehensive survey package comprised the newly translated Arabic YAS [1], a section for gathering demographic information, and other established scales deemed necessary for assessing convergent and divergent validity. Before the participants could commence the survey, an electronic informed consent form was explicitly presented to all prospective participants. This form clearly outlined the study’s objectives and the nature of participation and unequivocally stated their right to withdraw from the study at any point without incurring any consequences. The questionnaire link was broadly disseminated across popular social media platforms, replicating the successful data collection strategies employed in prior regional studies.

### Adaptation and translation processes

The translation and cultural adaptation of the YAS into Arabic rigorously adhered to the internationally recognized “forward-backward-forward technique”. This methodical, multi-step process was necessary to ensure both the linguistic accuracy and conceptual equivalence of the scale within the Arabic cultural context, aligning with the Principles of Good Practice for Translation and Cultural Adaptation [32]. The translation procedure included the following distinct stages:

1. Preparation: Initially, the research team meticulously prepared the questionnaire. This involved carefully selecting and integrating the appropriate scales to ensure that the study’s aims were effectively addressed.
2. Forward translation and reconciliation: The original English version of the YAS underwent independent translation into Arabic. This crucial step was performed by a specialized professional translator and two subject matter experts who possessed a profound understanding of both English and Arabic. A subsequent reconciliation process was then conducted to synthesize these independent translations into a single, cohesive Arabic version.
3. Back translation: Following the forward translation, the reconciled Arabic version was translated back into English by a different professional translator who was entirely blinded to the original English version of the scale. This back-translation served as a critical quality control step, meticulously verifying that the translated content maintained fidelity to the original in terms of meaning, tone, and wording.
4. Harmonization: A collaborative harmonization phase was undertaken by the designated experts and the research team. During this stage, the back-translated English version was systematically compared against the original English questionnaire. Any identified discrepancies, inconsistencies, or areas necessitating cultural adjustment were thoroughly discussed, resolved, and necessary modifications were implemented to achieve robust theoretical equivalence.
  The reconciled and back-translated versions were reviewed by a panel of bilingual experts who were native to, or highly familiar with, the four target countries (Bahrain, Saudi Arabia, Jordan, Tunisia). The panel ensured that the final Arabic version used neutral Modern Standard Arabic (MSA) wording that is widely comprehensible across regions, avoiding dialect-specific expressions. MSA is a standardized, formal variety of Arabic that is universally understood and used in written communication, education, media, and formal contexts across the Arab world. While formal cognitive debriefing interviews or dedicated pilot tests with target respondents were not conducted, the expert review focused on linguistic clarity, conceptual equivalence, and cultural appropriateness.
5. Proofreading and finalization: The conclusive Arabic version of the questionnaire was established through a rigorous proofreading process. This final review, involving both the researchers and experts, meticulously scrutinized all aspects of the scale, including grammar, syntax, clarity, and overall coherence, thereby guaranteeing the highest quality of the translated instrument.

### Sample size

We targeted a minimum sample size of *N* = 1,000, which is widely regarded in the psychometric literature as providing a complete or excellent level of adequacy for factor analysis in scale validation studies, particularly for models of low to moderate complexity, such as the unidimensional 6-item YAS examined here [33, 34]. Classic grading systems for factor analytic sample sizes classify *N* = 1,000 + as excellent, offering robust statistical power, stable parameter estimates, a low risk of convergence issues, and minimal bias even under nonideal conditions [33, 34]. This threshold substantially exceeds common rules of thumb such as *N* ≥ 300 for simple models or 5–10 observations per estimated parameter, yielding approximately 200–400 minimum cases for a 6-item CFA with approximately 12 parameters, and aligns with recommendations that larger samples enhance the precision of fit indices, reliability coefficients, and invariance testing [35].

### Measures

The central instrument utilized for this study was the YAS, which underwent translation and validation. The YAS is a 6-item psychometric tool developed based on Griffiths’ component model of addiction, assessing aspects such as salience, mood modification, tolerance, withdrawal, conflict, and relapse. Participants responded to each statement using a 5-point Likert scale, with responses ranging from “1 = Never” to “5 = Always”. A higher YAS score indicates a greater addiction severity [1].

To comprehensively establish the convergent and divergent validity of the Arabic YAS, additional validated Arabic scales were administered concurrently. Based on successful prior validation efforts for psychological constructs in the region, the following measures were included, as indicated by their presence in the correlation analyses:

*Insomnia Severity Index (ISI)* [36].

The ISI was used to assess sleep problems. This brief self-report instrument evaluates the nature, severity, and impact of insomnia symptoms. It consists of seven items evaluating difficulties with sleep onset, sleep maintenance, early morning awakening, satisfaction with sleep patterns, interference with daily functioning, noticeability of sleep problems, and distress caused by sleep difficulties. Respondents rate each item on a 5-point Likert scale, with higher total scores indicating greater severity of insomnia symptoms. The validated Arabic version of ISI was utilized in this research [37].

*Modified Yale Food Addiction Scale (mYFAS)* [38].

The mYFAS 2.0 was used to assess addictive eating behaviors. This self-report instrument was developed to assess addictive-like eating behaviors based on the diagnostic criteria for substance use disorders described in the DSM-5. The scale evaluates symptoms such as loss of control over eating, persistent desire to cut down, continued overeating despite negative consequences, and withdrawal-like experiences associated with highly palatable foods. Items are rated on a frequency scale, and higher scores indicate greater severity of food addiction–related behaviors. The validated Arabic version of mYFAS was utilized in this study [39].

*Depression Anxiety Stress Scales (DASS-21)* [40].

The DASS-21 was used to assess psychological distress. This widely used self-report instrument measures three related dimensions including depression, anxiety, and stress. The scale comprises 21 items divided into three subscales (seven items each) assessing symptoms such as low mood, physiological arousal, and tension or irritability. Respondents indicate the extent to which each statement applied to them during the past week using a 4-point Likert scale, with higher scores reflecting greater emotional distress. The validated Arabic version of DASS-21 was used in this study [41].

*Bergen Social Media Addiction Scale (BSMAS)* [22].

The BSMAS was used to assess general SMA. It is a brief instrument developed to assess problematic and potentially addictive use of social media platforms. The scale consists of six items reflecting the core components of behavioral addiction (i.e., salience, mood modification, tolerance, withdrawal, conflict, relapse) adapted specifically to social media use. Respondents rate the frequency of these experiences on a Likert-type scale, with higher scores indicating greater risk of problematic social media engagement. The validated Arabic version of BSMAS was used in this research [42].

To evaluate the temporal stability of the Arabic YAS, a convenience subsample of 200 participants (approximately 20% of the total sample) was invited to complete the scale a second time after a two-week interval. Participants were recruited from the main online survey pool on a voluntary basis during the final week of initial data collection. Eligibility required completion of the baseline assessment and provision of consent for follow-up contact. The retest was administered via the same online platform (identical survey link and instructions) to ensure consistency of mode and format. The retest participants received an automated email invitation exactly 14 days after their initial submission, with one reminder sent after 3 days if no response was received.

### Statistical analysis

All the statistical analyses were performed using specialized software packages, namely, STATA 17 version MP and R, for statistical computing 4.5.1. The significance level *p* was set at 0.05. Prior to any inferential analyses, thorough data integrity checks were conducted, involving rigorous screening procedures to identify and manage missing values, detect outliers, and flag potential automated or careless responses. Assumptions for statistical tests, including normality (via skewness and kurtosis indices) and multicollinearity (using Variance Inflation Factors), were assessed.

Participants were recruited via convenience sampling through social media platforms from four countries: Bahrain (*n* = 167), Jordan (*n* = 382), Saudi Arabia (*n* = 379), and Tunisia (*n* = 206), yielding a total sample of 1,134. To examine cross-national equivalence of the Arabic YAS, country-specific descriptive statistics for the total score were computed, and measurement invariance was evaluated at both the scale and item levels. Scale-level equivalence was assessed via one-way analysis of variance (ANOVA) on YAS Total scores across countries, supplemented by Levene’s test for homogeneity of variances and the non-parametric Kruskal–Wallis test. Item-level equivalence was tested for differential item functioning (DIF) using ordinal logistic regression (Zumbo’s approach), with YAS total score as the matching variable and country as the grouping factor (Jordan as reference). Uniform DIF was examined via the main effect of country, and non-uniform DIF via the country × total score interaction. Statistical significance was evaluated against a Bonferroni-corrected *α* = 0.008 (0.05/6 items).

The comprehensive psychometric evaluation involved several key statistical procedures:

Confirmatory factor analysis (CFA): To definitively assess the underlying factor structure of the Arabic YAS, a CFA was performed. This analysis systematically compared the fit of the hypothesized model against conventional indices, including the normed model chi-square (*χ²*/df), the Root Mean Square Error of Approximation (RMSEA), the Tucker-Lewis Index (TLI), and the Comparative Fit Index (CFI). Target values for good model fit (e.g., *χ²*/df ≤ 5, RMSEA ≤ 0.08, and CFI/TLI ≥ 0.95) guided interpretation. Additionally, the Average Variance Extracted (AVE) was computed to provide further evidence for convergent validity at the construct level (values of 0.5 or more were considered adequate). Prior to the main CFA on the full sample (*N* = 1,134), an exploratory factor analysis (EFA) was conducted on a random split subsample of the first 200 cases to preliminarily examine the factor structure and confirm alignment with the original unidimensional model.

Reliability analysis: The internal consistency of the Arabic YAS was assessed by calculating coefficient omega (*ω* ), Cronbach’s alpha (*α*), composite reliability (CR), Guttman’s lambda-2 coefficient (*λ₂*), and Guttman’s lambda-6 coefficient (λ₆). Acceptable reliability was indicated by values exceeding 0.70, with values above 0.80 considered good.

Test-retest reliability was quantified using the intraclass correlation coefficient (ICC) for absolute agreement under a two-way mixed-effects model (ICC [43]), with 95% confidence intervals (CIs) reported.

Measurement invariance: To ensure that the YAS functioned equivalently across different subgroups, multi-group confirmatory factor analysis (MGCFA) was conducted to examine measurement invariance of the scale scores. This involved assessing configural, metric, and scalar levels of invariance for three grouping variables: sex, age group, and weekly YouTube use. Configural invariance tested whether the single-factor structure held across groups with all parameters freely estimated; metric invariance constrained factor loadings to be equal across groups to evaluate whether items related to the latent construct with the same strength; and scalar invariance further constrained item intercepts (or thresholds) to equality, allowing valid comparisons of latent means. Given the sample size (*N* = 1,134), which can make the *χ²* difference test overly sensitive, invariance at each level was evaluated primarily using change-in-fit criteria. Invariance was supported when the decrease in CFI was ≤ 0.010, the increase in RMSEA was ≤ 0.015, and the increase in SRMR was ≤ 0.010, or ≤ 0.030 for the metric step according to some guidelines [44, 45]. The subgroups examined were sex (Female: *n* = 606, 53%; Male: *n* = 528, 47%), age group within the 18–25 range (18–21 years: *n* = 928, 82%; 22–25 years: *n* = 206, 18%), and weekly YouTube use (No: *n* = 557, 49%; Yes: *n* = 577, 51%). For age grouping the two-group split was selected to distinguish early emerging adulthood typically characterized by higher educational dependence, identity exploration, and transition from adolescence from later young adulthood often marked by greater independence, workforce entry, and stabilization, a common division in developmental psychology and young adult health/behavior research [29].

Convergent and divergent validity: The scores from the Arabic YAS were correlated with scores from the established measures of ISI, mYFAS, DASS-21, and BSMAS. The inter-correlation between the YAS factor and these other measures was scrutinized to confirm its distinct yet interrelated nature. Pearson product-moment correlation coefficients were used to assess convergent and divergent validity. However, as a sensitivity analysis, Spearman rank-order correlations were additionally performed to account for the ordinal response format of the scales and potential deviations from normality.

Item response theory (IRT) analysis: IRT analysis was conducted using the Partial Credit Model (PCM), a polytomous Rasch model suitable for ordered categorical (Likert-type) responses on the six-item YAS. The PCM was estimated via Marginal Maximum Likelihood Estimation (MMLE) with delta-tau parameterization in the eRm package in R [46]. This model was selected to allow item-specific threshold (step) structures, which is appropriate for behavioral scales where category difficulties may vary across items. Item fit was evaluated using inlier-sensitive (infit) and outlier-sensitive (outfit) mean-square (MnSq) statistics, with values between 0.5 and 1.5 considered acceptable for good fit to the Rasch model [47, 48]. Threshold parameters were inspected for monotonic ordering to confirm proper functioning of the response categories. Person separation reliability was calculated to assess measurement precision [47, 48]. Item difficulty parameters (in logits), standard errors, fit statistics, and threshold estimates were reported to evaluate the scale’s hierarchical structure, targeting of the latent trait (problematic YouTube use), and overall psychometric performance in the whole sample.

### Ethical considerations

Prior to the commencement of any data collection, full ethical approval was sought and obtained from the Institutional Review Board (IRB) of the Psychiatric Hospital, Government Hospitals, Bahrain (GH/PSY/REC/2024/165; decision date: 26 December 2024). Participation in this study was entirely voluntary, and comprehensive informed consent was electronically secured from all eligible participants. The utmost confidentiality and anonymity of all participant data were rigorously maintained throughout the entire duration of the study.

## Results

### Descriptive statistics

The descriptive statistics for all the study variables are presented in Table 1. The sample consisted of 1,134 participants with a mean age of 19.26 years (SD = 2.07), ranging from 18 to 25 years. The age distribution showed positive skewness (1.42), indicating that most participants were clustered toward the younger end of the age range.

**Table 1:** Descriptive statistics of the study variables (*N* = 1,134)

Variable	Mean	Median	SD	IQR	Minimum	Maximum	Skewness	Kurtosis
Age (Years)	19.26	18.00	2.07	2.00	18.00	25.00	1.42	0.65
YAS Total	16.13	16.00	5.03	7.00	6.00	30.00	0.08	-0.48
ISI Total	11.38	11.00	4.77	6.75	1.00	26.00	0.16	-0.45
mYFAS Total	15.49	12.00	11.91	17.00	0.00	57.00	0.98	0.40
DASS-21 Total	16.37	16.00	6.90	9.00	1.00	45.00	0.45	0.82
Depression	6.03	6.00	2.99	4.00	0.00	18.00	0.42	0.45
Anxiety	3.45	3.00	2.59	3.00	0.00	17.00	1.16	1.98
Stress	6.88	7.00	2.97	4.00	0.00	20.00	0.28	0.66
BSMAS Total	15.47	14.00	4.25	4.00	6.00	30.00	1.15	1.91

SD= Standard Deviation IQR= Interquartile Range; Age (Years) = Chronological Age in Years; YAS Total = YouTube Addiction Scale Total Score; ISI Total = Insomnia Severity Index Total Score; mYFAS Total = Modified Yale Food Addiction Scale Total Score; DASS-21 Total = Depression Anxiety Stress Scales Total Score; Depression = Depression Subscale Score; Anxiety = Anxiety Subscale Score; Stress = Stress Subscale Score; BSMAS Total = Bergen Social Media Addiction Scale Total Score

Regarding behavioral addictions and problematic behaviors, participants demonstrated varying levels of engagement across different domains. YAS showed a mean of 16.13 (SD = 5.03) with a relatively normal distribution (skewness = 0.08, kurtosis = -0.48), suggesting balanced representation across addiction severity levels. Social media addiction scores (BSMAS) averaged 15.47 (SD = 4.25) with positive skewness (1.15), indicating that most participants exhibited lower levels of social media addiction with fewer individuals showing severe addiction patterns.

Sleep disturbances, as measured by the ISI, showed a mean score of 11.38 (SD = 4.77) with minimal skewness (0.16), suggesting normal distribution of sleep difficulties across the sample. The median score of 11.00 indicates that half of the participants experienced mild to moderate insomnia symptoms.

The mYFAS demonstrated considerable variability with a mean of 15.49 (SD = 11.91) and a wide range from 0 to 57. The positive skewness (0.98) and substantial interquartile range (IQ*r =* 17.00) indicate that while most participants reported minimal food addiction symptoms, a subset exhibited notably higher levels of problematic eating behaviors.

The mental health indicators revealed moderate levels of psychological distress across the sample. The DASS-21 total score averaged 16.37 (SD = 6.90), with depression subscale scores (M = 6.03, SD = 2.99), anxiety subscale scores (M = 3.45, SD = 2.59), and stress subscale scores (M = 6.88, SD = 2.97). The anxiety subscale showed the highest positive skewness (1.16) and kurtosis (1.98), suggesting that while most participants reported low anxiety levels, a minority experienced significantly elevated anxiety symptoms.

Country-specific descriptive statistics for the YAS total score revealed comparable means and variability across sites: Bahrain (*n* = 167, M = 15.93, SD = 4.92), Jordan (*n* = 382, M = 16.00, SD = 5.11), Saudi Arabia (*n* = 379, M = 16.28, SD = 5.04), and Tunisia (*n* = 206, M = 16.27, SD = 4.98). One-way ANOVA indicated no significant differences in mean scores across countries, F(3, 1130) = 0.341, *p =* 0.79. Levene’s test confirmed homogeneity of variances (*p =* 0.97), and the Kruskal–Wallis test corroborated the non-significant result (H = 1.03, *p =* 0.79). At the item level, ordinal logistic regression DIF analyses showed no evidence of uniform or non-uniform DIF for any of the six items (all p-values > 0.30 for uniform DIF and > 0.52 for non-uniform DIF, all exceeding the Bonferroni-corrected threshold of 0.008).

The initial EFA of the first 200 cases confirmed a clear unidimensional structure as follows: single factor eigenvalue > 4.0, all items loading > 0.50, no secondary factor, and variance explained > 65%. The hypothesized unidimensional model on the full sample provided excellent fit. Alternative models, e.g., bifactor and two-factor exploratory groupings, were considered but not pursued in the main analyses due to a lack of theoretical support and no improvement indicated in preliminary checks.

### Confirmatory factor analysis of the YouTube addiction scale

All factor loadings were statistically significant (*p <* 0.001) and ranged from 0.55 to 0.73 (standardized), indicating strong relationships between the observed items and the underlying factor. CFA results are presented in Table 2. Item 4 demonstrated the highest factor loading (λ = 0.73, SE = 0.03, z = 25.64), followed by Item 1 (λ = 0.68, SE = 0.04, z = 23.19) and Item 2 (λ = 0.65, SE = 0.04, z = 22.06). The weakest, yet still acceptable, factor loading was observed for Item5 (λ = 0.55, SE = 0.04, z = 18.02). All residual variances were significant (*p <* 0.001), ranging from 0.47 to 0.70 (standardized), indicating that substantial portions of item variance remained unexplained by the common factor. The model fit indices demonstrated excellent fit across multiple indicators. The chi-square test was significant (*χ²* = 40.66, df = 9, *p <* 0.001), which is expected given the large sample size. The comparative fit indices showed excellent fit: CFI = 0.99, TLI = 0.98, and IFI = 0.99, all of which substantially exceeded the conventional threshold of 0.90 for acceptable fit. The NFI (0.98) and RFI (0.97) also demonstrated excellent fit levels.

**Table 2:** Confirmatory factor analysis and reliability analysis of the Arabic version of the YouTube addiction scale (6-items) (*N* = 1,134)

Factor loadings						Residual variances			
Factor	Indicator	Value	SE	z-value	*p*-value	Value	SE	z-value	*p*-value
Factor 1	Item1	0.68	0.04	23.19	< 0.001	0.54	0.07	18.61	< 0.001
	Item2	0.65	0.04	22.06	< 0.001	0.58	0.06	19.34	< 0.001
	Item3	0.60	0.04	20.00	< 0.001	0.64	0.07	20.71	< 0.001
	Item4	0.73	0.03	25.64	< 0.001	0.46	0.07	17.16	< 0.001
	Item5	0.55	0.04	18.02	< 0.001	0.70	0.06	21.25	< 0.001
	Item6	0.61	0.03	20.76	< 0.001	0.62	0.06	19.90	< 0.001

SE = Standard Error. Method: Confirmatory factor analysis was conducted using Weighted Least Squares; reported results are standardized. Factor 1 = Items1-6. Model Fit Statistics Chi-square Test Baseline model: *χ²* = 2699.50, df = 15, *p <* 0.001 Factor model: *χ²* = 40.66, df = 9, *p <* 0.001. Fit Indices Comparative Fit Index (CFI): 0.99 Tucker-Lewis Index (TLI): 0.98 Bentler-Bonett Non-normed Fit Index (NNFI): 0.98 Bentler-Bonett Normed Fit Index (NFI): 0.98 Parsimony Normed Fit Index (PNFI): 0.59 Bollen’s Relative Fit Index (RFI): 0.97 Bollen’s Incremental Fit Index (IFI): 0.99 Relative Noncentrality Index (RNI): 0.99 Information Criteria Log-likelihood: Not available Number of free parameters: 12 Akaike (AIC): Not available Bayesian (BIC): Not available Sample-size adjusted Bayesian (SSABIC): Not available Other Fit Measures Root Mean Square Error of Approximation (RMSEA): 0.06 RMSEA 90% CI: [0.04, 0.07] RMSEA p-value: 0.27 Standardized Root Mean Square Residual (SRMR): 0.04 Hoelter’s Critical N (*α* = 0.05): 472.50 Hoelter’s Critical N (*α* = 0.01): 604.79 Goodness of Fit Index (GFI): 0.99 McDonald Fit Index (MFI): 0.99 Expected Cross Validation Index (ECVI): 0.06 Kaiser-Meyer-Olkin (KMO) Test YT1: 0.82 YT2: 0.82 YT3: 0.89 YT4: 0.84 YT5: 0.85 YT6: 0.83 Overall KMO: 0.84, Parameter Estimates Average Variance Extracted (AVE) Factor 1: 0.41

The RMSEA of 0.06 (90% CI [0.04, 0.07]) indicated good fit, falling well below the conventional threshold of 0.08 for acceptable fit, with the p-value of 0.27 suggesting no significant misfit. The SRMR (0.04) was excellent, falling well below the 0.08 threshold. Additional fit indices including the Goodness of Fit Index (GFI = 0.99) and McDonald Fit Index (MFI = 0.99) further supported the model’s excellent fit.

The AVE for the single factor was 0.41, falling just below the conventional threshold of 0.50 for adequate convergent validity. This suggests that while the factor structure is well-supported, there may be opportunities to enhance the scale’s convergent validity.

The factor model demonstrated substantial improvement over the baseline independence model, with the chi-square difference being highly significant (Δ*χ²* = 2658.84, Δdf = 6, *p <* 0.001; calculated as 2699.50–40.66). The Kaiser-Meyer-Olkin (KMO) test indicated excellent sampling adequacy with an overall KMO of 0.84, with individual item KMO values ranging from 0.82 to 0.89.

### Internal consistency reliability and test-retest reliability

The YAS demonstrated good internal consistency across multiple reliability indices (Table 3). McDonald’s omega (*ω* = 0.81, 95% CI [0.79, 0.82]), Cronbach’s alpha (*α* = 0.80, 95% CI [0.78, 0.82]), and composite reliability (C*r =* 0.80, 95% CI [0.78, 0.82]) are all equal to or exceed the 0.800 threshold for good reliability. Guttman’s lambda-2 (*λ₂* = 0.80, 95% CI [0.79, 0.82]) and lambda-6 (λ₆ = 0.78, 95% CI [0.77, 0.80) coefficients similarly indicated acceptable to good internal consistency.

**Table 3:** YouTube addiction scale reliability statistics and item-total correlations (*N* = 1,134)

Reliability	McDonald’s ω	Cronbach’s α	Guttman’s λ₂	Guttman’s λ₆
Overall Scale	0.81 [95% CI 0.79–0.82]	0.80 [95% CI 0.78–0.82]	0.80 [95% CI 0.79–0.82]	0.78 [95% CI 0.77–0.80]
If Item Deleted				
Item1	0.77	0.76	0.76	0.73
Item2	0.77	0.77	0.77	0.74
Item3	0.78	0.78	0.78	0.75
Item4	0.76	0.75	0.76	0.73
Item5	0.79	0.79	0.79	0.76
Item6	0.78	0.78	0.78	0.74

McDonald’s *ω* = McDonald’s omega coefficient; Cronbach’s *α* = Cronbach’s alpha coefficient; Guttman’s *λ₂* = Guttman’s lambda-2 coefficient; Guttman’s λ₆ = Guttman’s lambda-6 coefficient

Item-total correlation analysis (via “if item deleted”) revealed that all items contributed positively to scale reliability. Removing any individual item decreased overall reliability across measures. For McDonald’s omega, coefficients after deletion ranged from 0.76 (Item 4) to 0.79 (Item 5). Similar patterns appeared for Cronbach’s alpha (ranging from 0.75 for Item 4 to 0.79 for Item 5). Items 4 and 1 showed the strongest contributions to reliability, as their removal resulted in the largest drops (e.g., *ω* = 0.76 and *α* = 0.75–0.76). Item 5 showed the smallest contribution, with the least reduction in reliability upon removal (*ω* = 0.79, *α* = 0.79), though deletion still meaningfully lowered coefficients.

These findings support the retention of all six items and confirm the scale’s adequate psychometric properties for measuring YouTube addiction behaviors.

Additionally, a subsample of 200 participants took the test twice, separated by two weeks, and the ICC was found to be 0.87 [95% CI 0.84–0.89].

### Item response theory analysis

Model diagnostics supported good fit to the data (*N* = 1,134). Item difficulty parameters (δ) ranged from − 2.27 logits (Item 2, easiest/most endorsed) to -0.48 logits (Item 6, most difficult/least endorsed), indicating relatively easy targeting overall in this young adult sample (Table 4). The spread of ~ 1.8 logits suggests reasonable coverage of the latent trait continuum (problematic YouTube use/addiction), though with a mild floor effect (items clustered toward the easier end). Person reliability was 0.78, indicating adequate precision for group-level comparisons and moderate individual-level discrimination, consistent with the CFA reliability estimate (*ω* = 0.80).

**Table 4:** Item response theory of the YouTube addiction scale (*N* = 1,134)

Item statistics of the rating scale model					Delta-tau parameterization of the partial credit model				
Item	Measure	S.E.	Infit	Outfit	1	2	3	4	5
Item1	-2.06	0.03	0.94	0.94	-31.00	6.58	7.14	8.17	9.14
Item2	-2.27	0.03	0.97	0.98	-30.60	6.22	7.27	8.10	9.05
Item3	-2.02	0.03	1.12	1.13	-30.80	6.78	7.13	7.96	8.94
Item4	-1.32	0.03	0.91	0.88	-32.80	6.92	7.88	8.56	9.44
Item5	-1.28	0.03	1.15	1.17	-32.50	6.85	7.88	8.46	9.27
Item6	-0.48	0.04	0.96	0.86	-32.60	6.93	8.00	8.40	9.24

The analysis conducted using the Polytomous Rasch Model (Partial Credit Model) with delta-tau parameterization via Marginal Maximum Likelihood Estimation (MMLE). The eRm R package was used for analysis and person-item mapping. Person reliability = 0.78, indicating adequate measurement precision. Measure = item difficulty parameter in logits (more negative values indicate easier items); S.E. = standard error of the item difficulty estimate; Infit = information-weighted mean square statistic; Outfit = outlier-sensitive mean square statistic

Infit and outfit mean-square statistics ranged from 0.86 to 1.17, all within commonly accepted bounds (0.7–1.3 for polytomous items), confirming good item fit with no evidence of serious misfit or multidimensionality. Threshold parameters (step difficulties) increased monotonically within each item (e.g., tau1 < tau2 < tau3 < tau4 < tau5 across all items), demonstrating ordered response categories and proper functioning of the 5-point rating scale with no category disordering or reversals.

Unidimensionality was supported by the prior CFA (excellent single-factor fit and full scalar invariance) and by inspection of residual correlations (Yen’s Q3 statistic and pairwise standardized residual correlations remained below |0.20–0.30| thresholds in preliminary diagnostics, indicating no substantial local dependence). Targeting was adequate, as the item difficulties aligned reasonably well with the person ability distribution, enabling reliable differentiation along the trait continuum.

### Correlation analyses

Pearson correlation analyses revealed significant associations between YouTube addiction and all measured variables (Table 5). YouTube addiction showed the strongest correlation with SMA (*r =* 0.66, *p <* 0.001), followed by overall psychological distress (*r =* 0.40, *p <* 0.001) and food addiction (*r =* 0.32, *p <* 0.001). Among the DASS-21 subscales, YouTube addiction correlated most strongly with stress (*r =* 0.35, *p <* 0.001) and depression (*r =* 0.34, *p <* 0.001) and moderately with anxiety (*r =* 0.28, *p <* 0.001). The correlation with insomnia severity was moderate (*r =* 0.26, *p <* 0.001).

**Table 5:** Correlation analyses between study variables (*N* = 1,134)

Variable	1	2	3	4	5	6	7	8
1. YAS Total	—							
2. ISI Total	0.26** 0.24**	—						
3. mYFAS Total	0.32** 0.33**	0.07* 0.05	—					
4. DASS-21 Total	0.40** 0.39**	0.08** 0.07*	0.18** 0.19**	—				
5. Depression	0.34** 0.34**	0.07** 0.05	0.15* 0.17**	0.83** 0.83**	—			
6. Anxiety	0.28** 0.27**	0.06** 0.06*	0.14* 0.14**	0.77** 0.74**	0.46** 0.45**	—		
7. Stress	0.35** 0.33**	0.07** 0.06*	0.14** 0.15**	0.82** 0.81**	0.52** 0.51**	0.45** 0.44**	—	
8. BSMAS Total	0.66** 0.70**	0.15** 0.12**	0.24** 0.25**	0.30** 0.32**	0.26** 0.29**	0.22** 0.22**	0.25** 0.26**	—

Pearson product-moment correlations are shown in the upper row of each cell; Spearman rank-order correlations are shown in the lower row. YAS Total = YouTube Addiction Scale Total Score; ISI Total = Insomnia Severity Index Total Score; mYFAS Total = Modified Yale Food Addiction Scale Total Score; DASS-21 Total = Depression Anxiety Stress Scales Total Score; Depression = Depression Subscale Score; Anxiety = Anxiety Subscale Score; Stress = Stress Subscale Score; BSMAS Total = Bergen Social Media Addiction Scale Total Score. * *p <* 0.05, ** *p <* 0.001

SMA demonstrated significant positive correlations with all the variables, with the strongest associations observed with YouTube addiction and food addiction (*r =* 0.24, *p <* 0.001). Food addiction showed weak to moderate correlations with the psychological distress variables (*r =* 0.14–0.18, *p <* 0.05–0.001) but a minimal association with insomnia (*r =* 0.07, *p <* 0.05). The DASS-21 subscales showed strong intercorrelations (*r =* 0.45–0.83, *p <* 0.001), confirming the shared variance in psychological distress symptoms.

All Spearman rank-order correlations are presented in Table 5. All correlations remained statistically significant and highly similar in magnitude between the two methods, confirming the robustness of the validity findings.

## Discussion

The present study successfully translated and rigorously validated the 6-item YAS [1] for use within Arabic-speaking young and emerging adult populations, addressing a crucial gap in culturally tailored assessment tools for this widely consumed digital platform. Our findings provide robust psychometric evidence for the Arabic YAS, supporting its utility for researchers and mental health professionals in Arab societies.

The consistent unidimensional structure across the original validation, our preliminary EFA on subsample cases, full-sample CFA, and independent adaptations (Turkish [49], Malay [50], Indonesian [51]) supports treating the six YAS items as indicators of a single latent YouTube addiction construct, in line with how brief component-model scales are typically operationalized. While Griffiths’ model conceptualizes six components [52], these co-occur as a unified syndrome in empirical brief measures. Future studies with longer item pools or multi-item per component designs could revisit potential multidimensionality of the construct.

The high prevalence of social media and YouTube engagement in the Arab world further underscores the importance of the present validation. With internet penetration reaching 77%, Arab youth spend over 3.5 h daily online and maintain an average of 8.4 social media accounts. In addition, nearly 74% report difficulty disconnecting, and 61% acknowledge negative effects on mental well-being [25, 26]. The availability of a validated and culturally appropriate tool, such as the Arabic YAS, is particularly timely. This instrument now enables researchers and clinicians across the region to systematically quantify the burden of problematic YouTube use and its intersection with the regional patterns of digital addiction documented in the Arab youth surveys.

A limitation of the current Rasch analysis is that all item difficulty parameters were negative (ranging from − 2.27 to − 0.48 logits), indicating that the YAS items were relatively easy to endorse in this young adult sample and thus primarily target the lower-to-moderate range of the latent trait (problematic YouTube use). This suggests suboptimal coverage of the upper end of the severity continuum (severe dependence), which may limit the scale’s sensitivity to detect high levels of addiction in clinical or high-risk populations. Based on the IRT analysis, the item hierarchy reveals a clear severity gradient in YouTube addiction behaviors [1, 49]. While the scale demonstrated adequate targeting and reliability for the present non-clinical sample (person reliability = 0.78), future revisions could benefit from including more difficult items (e.g., capturing severe withdrawal, conflict, functional impairment) to better span the full spectrum of YouTube addiction severity and enhance the instrument’s utility across diverse severity levels.

The scale also showed strong internal consistency, with most internal consistency coefficients ≥ 0.80 exceeding the conventional threshold for good reliability [53]. This high internal consistency suggests that the items of the Arabic YAS reliably cohere to measure YouTube addiction severity. Furthermore, full scalar measurement invariance was supported across sex and weekly YouTube use categories, indicating that the YAS structure, item-factor relationships, and item intercepts/thresholds are equivalent for females and males, as well as for participants who do versus do not use YouTube weekly. This supports the valid comparison of scale scores and latent means across these subgroups without bias.

The AVE for the single factor was 0.41, slightly below the conventional 0.50 threshold for strong convergent validity. However, all standardized factor loadings were significant and mostly > 0.60, Cronbach’s *α* and McDonald’s *ω* exceeded 0.80, and overall model fit was excellent, supporting acceptable convergent validity in the context of this brief scale. This pattern is not uncommon in short behavioral addiction scales [54, 55] and does not undermine the instrument’s utility as a dimensional screening tool for problematic YouTube use [1].

The successful psychometric validation of the Arabic YAS is consistent with Griffiths’ component model of addiction [1], as the six items showed strong unidimensional loadings and excellent fit without evidence of separable facets in this Arabic-speaking sample. This pattern aligns closely with findings from the original English validation and subsequent adaptations (Turkish [49], Malay [50], Indonesian [51]), suggesting that the core symptoms of salience, mood modification, tolerance, withdrawal, conflict, and relapse cohere as a unified construct of problematic YouTube use even in a culturally and linguistically distinct context. Furthermore, no Arabic-specific deviations from the model were observed, further supporting its cross-cultural robustness.

Our correlation analyses underscored the convergent and divergent validity of the Arabic YAS. As hypothesized, higher YAS scores were significantly and positively correlated with increased levels of general social media addiction (BSMAS). This association has also been reflected in previous literature. Participants in earlier studies with higher levels of YouTube addiction had significantly greater general social media addiction [1, 51].

Moreover, the Arabic YAS showed significant positive correlations with measures of psychological distress, including ISI, the mYFAS, and the DASS-21 total and subscales. These findings resonate with broader literature linking excessive social media and internet use to diminished work performance, less healthy social relationships, sleep disturbances, reduced life satisfaction, and heightened feelings of jealousy, anxiety, and depression [4, 15, 56, 57]. The observed correlation between higher YouTube addiction and greater psychological distress aligns with previous studies that have identified social anxiety as a significant predictor and exacerbator of online addiction [4, 15, 56, 57].

### Implications and future directions

The ability of the YAS to classify individuals into distinct subgroups based on addiction severity further supports its clinical utility. Previously latent class analysis identified two subgroups: one with higher YouTube addiction levels and another with lower levels [1]. The group with more severe YouTube addiction demonstrated significantly greater general SMA, higher psychological distress, and tended to be younger, while also spending more weekly hours on YouTube [1]. These findings are consistent with prior research linking increased YouTube addiction to adverse psychological outcomes and younger age demographics [1].

The validation of the YAS in Arabic is particularly important given the widespread consumption of online content and social media in Arab-speaking countries. This validated tool will enable mental health professionals to monitor the severity of individuals’ YouTube addiction and facilitate early intervention. It also opens avenues for cross-cultural comparisons of YouTube addiction prevalence and its correlates, offering deeper insights into how digital dependency manifests globally.

**Fig. 1:**
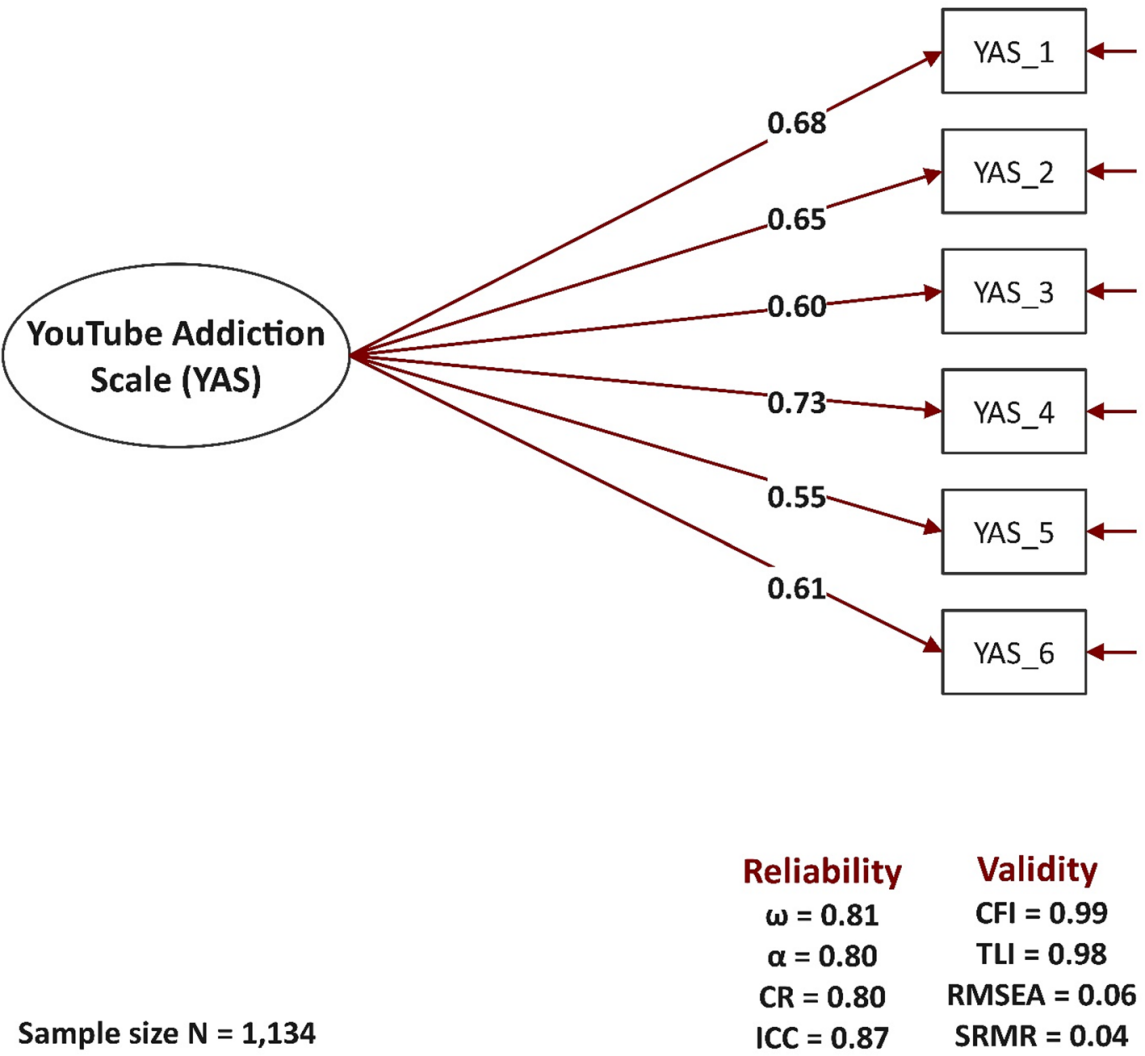
Path diagram of the confirmatory factor analysis for the Arabic YouTube Addiction Scale (YAS), including standardized factor loadings, model fit indices, reliability coefficients, and sample size (*N* = 1,134). YAS = YouTube Addiction Scale; Paths represent standardized factor loadings (all *p* < 0.001); ω = McDonald’s omega (ordinal reliability); α = Cronbach’s alpha; CR = Composite reliability; ICC = Intraclass correlation coefficient (test-retest reliability); CFI = Comparative Fit Index; TLI = Tucker–Lewis Index; RMSEA = Root Mean Square Error of Approximation; SRMR = Standardized Root Mean Square Residual; Sample size *N* = 1,134 (Arabic-speaking emerging and young adults)

Future research should build upon these findings. While the 6-item YAS is efficient, further studies could explore the dimensions identified in other YAS versions, such as the 22-item scale [2] which categorizes addiction into impaired control, decreased alternate pleasure, intense desire, and measures taken to reduce usage. Additionally, longitudinal studies are needed to establish causal relationships between YouTube addiction and its psychological consequences. Exploring the role of specific YouTube features, such as monetization programs (e.g., YPP), in contributing to addictive engagement would also provide valuable insights. Future studies should replicate the test–retest findings in larger and more diverse samples and examine responsiveness to change (Fig. 1).

### Strengths and limitations

The present study offers several notable strengths that enhance its contribution to the field of behavioral addiction research, particularly within Arabic-speaking populations. First, the study’s multi-country approach, encompassing Bahrain, Saudi Arabia, Jordan, and Tunisia, ensures broad cultural and linguistic representation, increasing the generalizability of the Arabic YAS across diverse Arab societies. This approach addresses the critical need for culturally tailored psychometric tools in a region where YouTube usage is highly prevalent. Second, the rigorous translation process, utilizing the forward-backward-forward technique, ensured linguistic accuracy and conceptual equivalence, aligning with international standards for scale adaptation. Third, the large sample size (*N* = 1,134) provided robust statistical power for psychometric analyses, including CFA and IRT, enhancing the reliability of the findings. Additionally, the inclusion of multiple validated scales (i.e., ISI, mYFAS, DASS-21, BSMAS) allowed for a comprehensive assessment of convergent and divergent validity, confirming the Arabic YAS as a distinct yet related measure of YouTube addiction. Finally, a key strength of this validation is the multi-country sample, enabling preliminary examination of cross-national applicability across four distinct Arabic-speaking contexts. The absence of significant differences in mean YAS Total scores (*p =* 0.79) and lack of differential item functioning (all DIF *p* > 0.008) indicate that the Arabic YAS functions equivalently in Bahrain, Jordan, Saudi Arabia, and Tunisia, supporting its use for comparative research and pooled analyses in the region. These results align with the choice of MSA for translation, which likely minimized dialectal influences on item interpretation. While the convenience sampling limits broader generalizability, the demonstrated measurement equivalence enhances confidence in the scale’s cross-cultural robustness for emerging adults. Future studies should replicate these invariance findings with probability-based samples, older age groups, and formal multi-group CFA to confirm scalar invariance across countries and further establish the Arabic YAS as a reliable tool for regional and potentially pan-Arab investigations of problematic YouTube use. Country-specific descriptive statistics for the YAS Total score revealed comparable means and variability across sites.

Despite its strengths, the study has several limitations specific to its design and execution. First, the sample was limited to emerging and young adults aged 18–25 years, reflecting the demographics of social media respondents, which restricts the generalizability of the findings to older adults. Future studies should consider recruiting a more diverse age range to investigate measurement invariance and addiction patterns across the entire adult lifespan. Second, the use of convenience sampling through social media channels may have introduced selection bias, potentially overrepresenting younger, tech-savvy individuals who are more active on platforms, such as YouTube, which could limit the generalizability of findings to older or less digitally engaged populations. Third, the cross-sectional design precludes establishing causality or temporal relationships between YouTube addiction and associated psychological outcomes (e.g., depression or insomnia). Fourth, although full scalar invariance was supported across age groups (18–21 years and 22–25 years), the relatively small size of the older subgroup (*n* = 206) approaches the lower boundary for reliable estimation using WLS methods. This may limit the precision of parameter estimates and the statistical power to detect subtle non-invariance in that group. Consequently, while the current findings indicate that the YAS functions equivalently across these age bands, future research with larger and more balanced samples across finer developmental gradations is recommended to further confirm the robustness of scalar invariance and to better capture potential age-related nuances in item responding or latent trait expression during emerging and young adulthood. A further delimitation is the exclusive reliance on self-report measures without corroboration from a gold-standard clinical interview or objective behavioral indicators, rendering the findings vulnerable to social desirability bias and self-presentation effects. Finally, test-retest reliability was assessed only on a small subsample (*n* = 200), which limits the generalizability of conclusions regarding the scale’s temporal stability across the full sample.

## Conclusions

The Arabic YAS is a psychometrically sound instrument for assessing the severity of problematic YouTube use among emerging adults in Arabic-speaking populations; it has a unidimensional structure, good internal consistency, adequate item fit via item response theory, and significant correlations with related constructs such as general social media addiction, psychological distress, food addiction, and insomnia. Given the cross-sectional design of this validation study, no causal inferences can be drawn regarding the directionality of associations between problematic YouTube use and psychological outcomes such as distress, sleep disturbances, or other behavioral domains. The observed correlations indicate co-occurrence but do not establish that problematic use leads to these difficulties or vice versa; longitudinal research is needed to clarify temporal relationships and potential bidirectional influences. Furthermore, the Arabic YAS functions as a dimensional measure of symptom severity rather than a diagnostic instrument—no clinical cutoffs, sensitivity/specificity metrics, or predictive validity evidence are yet available to support its standalone use for identifying clinically significant cases or guiding individual-level interventions.

While the scale shows promise for facilitating early identification of elevated risk in research and community settings, claims regarding direct clinical application, e.g., enabling targeted early intervention, remain preliminary and should be interpreted cautiously until future studies establish cutoff scores linked to functional impairment, test responsiveness to change, and real-world clinical utility. The present validation primarily advances measurement readiness for culturally relevant investigations of problematic YouTube use in the Arab region and highlights the need for prospective designs, broader age representations, and additional validity testing to strengthen its applied value.

## Data Availability

Data are available from the corresponding author upon reasonable request.
